# Private Equity Acquisition in Primary Care and Avoidable Hospitalizations

**DOI:** 10.1001/jamahealthforum.2026.1045

**Published:** 2026-05-08

**Authors:** Meehir N. Dixit, Alexander P. Philips, Amal N. Trivedi, Christopher Whaley, Yashaswini Singh

**Affiliations:** 1 Department of Health Services, Policy, and Practice, Brown University School of Public Health, Providence, Rhode Island; 2 Pritzker School of Medicine, The University of Chicago, Chicago, Illinois; 3 Warren Alpert School of Medicine, Brown University, Providence, Rhode Island

## Abstract

**Importance:**

Private equity (PE) is one form of corporate investment that has rapidly expanded into primary care, with more than 2400 primary care physicians becoming PE-affiliated since 2019. There are concerns that profit incentives associated with PE investment might be detrimental to care quality and patient outcomes.

**Objective:**

To examine changes in patient outcomes for the traditional Medicare (TM) population after primary care practices are acquired by PE firms and to identify any changes in patient composition.

**Design, Setting, and Participants:**

This economic evaluation used a stacked difference-in-differences analysis with a 20% Medicare Part B sample from 2016 to 2022. Medicare beneficiaries with PE-acquired primary care physicians were matched to control patients based on age, risk score, sex, race and ethnicity, state of residence, and dual-eligibility status. Statistical analysis was performed from November 2024 to February 2026.

**Exposure:**

Primary care practice acquisition by a PE firm, identified using PitchBook data.

**Main Outcomes and Measures:**

Primary outcomes at the patient-quarter level include number of all-cause hospitalizations, number of potentially avoidable hospitalizations for ambulatory-sensitive conditions, and number of emergency department (ED) visits. Secondary outcomes include measures of patient composition, including patient age, sex, race and ethnicity, and hierarchical condition category score.

**Results:**

The analysis included 24 397 beneficiaries with PE-acquired primary care physicians, matched to 121 939 control patients. The mean (SD) age was 74 (10) years, and 56% of patients were female. After PE acquisition, the number of all-cause ED visits decreased by 1.36% (95% CI, −2.72% to −0.14%) per patient-quarter relative to baseline. Considering various sensitivity tests, there were no significant changes to the probability of or number of potentially preventable hospitalizations or all-cause hospitalizations. Patient composition remained unchanged.

**Conclusions and Relevance:**

In this national study of traditional Medicare beneficiaries, PE acquisitions of primary care practices were not associated with meaningful short-term changes in acute care outcomes. Overall, findings contribute to policy discourse on understanding the role of PE investments in shaping care quality, suggesting heterogeneity in outcomes across health care settings.

## Key Points

**Question:**

Do acute care outcomes change after private equity acquisition of primary care practices?

**Findings:**

In this economic evaluation with a difference-in-differences analysis, private equity (PE) acquisition of primary care practices was not associated with clinically meaningful changes to all-cause or preventable hospitalizations. PE acquisitions were associated with a modest reduction in emergency department visits. There were no observed changes in patient composition in terms of demographics and patient risk.

**Meaning:**

PE acquisitions in primary care did not yield meaningful changes in all-cause or preventable hospitalizations for the traditional Medicare population; these findings highlight the heterogeneity in patient outcomes following PE acquisition across care settings.

## Teaser

This economic evaluation examines changes in patient outcomes and composition among patients with traditional Medicare after the acquisition of primary care practices by private equity firms.

## Introduction

In recent years, private equity (PE) firms have rapidly expanded into primary care, with more than 2400 primary care physicians (PCPs) estimated to be in PE-affiliated practices in 2019.^1^ The emergence of PE in health care has raised concerns that the profit incentives of PE may erode quality of care. Yet, other forms of physician ownership, including hospital- and payer-affiliated entities, are not immune from profit incentives.^1,2,3,4^ PE investments may provide financial resources to implement health information technology and participate in value-based contracts.^5,6^ At the same time, PE’s platform and add-on model of consolidation^7,8^ have resulted in higher negotiated prices^1,9,10,11,12^ and changes to utilization,^13,14,15^ with mixed outcomes for care quality.^16,17^ Importantly, while prior studies have found negative associations with patient outcomes and experiences following PE acquisition, evidence is mostly concentrated in the nursing home^18,19,20^ and hospital sectors,^21,22,23,24^ which may not generalize to physician practices.^25,26^ Whether PE acquisitions in primary care translate to measurable differences in patient outcomes, such as avoidable hospitalizations, remains a key unknown.

PE ownership may influence hospitalizations for ambulatory care–sensitive conditions (ACSCs) through several mechanisms. First, PE investments in health care have been associated with workforce reductions and turnover,^27,28,29^ which in turn can impair the timely management of chronic conditions. Second, PE-backed practices may shift focus toward more profitable services or higher-revenue patient populations,^13,15,30,31,32^ potentially shifting care away from preventive management. Conversely, PE capital infusion could enable investments in care management infrastructure, extended hours, or telehealth capabilities,^5^ though whether such changes translate into downstream improvements in patient outcomes remains unclear.

Understanding these dynamics is critical given hospitalizations for ACSCs are a key metric that signals gaps in timely, high-quality outpatient care.^33,34,35,36,37^ Among Medicare beneficiaries, avoidable hospitalizations for ACSCs account for $25.8 billion each year, roughly 3% of total program spending.^38^ In 2017 alone, more than 3.5 million adult inpatient stays—approximately 1 in every 8 admissions—were judged preventable, with $33.7 billion in hospital costs generated.^39^ Beyond the fiscal burden, preventable admissions expose patients to iatrogenic harm and strain inpatient capacity, making them a clinically intuitive target to improve the value of care.^35,40,41,42^

Traditional Medicare beneficiaries experience a disproportionate share of ACSC admissions, due to advanced age, multimorbidity, and complex social risk.^43,44^ Risk-standardized ACSC hospitalization rates vary nearly 2-fold across hospital service areas, underscoring wide geographic variation and opportunity for improvement.^45^ These patterns mirror long-standing socioeconomic and insurance-related disparities in avoidable hospitalizations documented from prior work.^46,47,48^ Because traditional Medicare covers a nationally representative, policy-relevant population and offers complete claims data, it remains an ideal lens through which to study potentially avoidable hospitalizations following PE acquisitions in primary care.

Using national traditional Medicare claims linked to a hand-collected database of PE transactions, we examine changes in patient outcomes after primary care practices were acquired by PE firms. Our aims were to (1) examine changes in outcomes sensitive to primary care quality and (2) identify any changes in patient composition.

## Methods

There were multiple steps involved in creating the analytic sample for this economic analysis, outlined below. Details are provided in the eMethods in Supplement 1. Brown University’s institutional review board approved this study with a waiver of informed consent given the use of deidentified data. We followed the Consolidated Health Economic Evaluation Reporting Standards (CHEERS) reporting guideline for economic analyses.

### Identifying Private Equity Acquisitions

We used multiple data sources to identify physicians in PE-acquired practices. PE acquisitions from 2016 to 2022 were identified using proprietary PitchBook Inc data, used by other studies to examine PE in health care.^9,10,11,13,49^ Each acquisition was manually verified and the list was expanded using press releases, industry reports, and current and archived physician practice websites.

### Identifying Physicians Affiliated With Practices

Physician owners of practices were identified using web searches for their National Provider Identifiers (NPIs), following validated methods.^10,11,14,28^ Acquisitions were linked to taxpayer identification numbers by matching the owner’s NPI, business name, and location to the Medicare Data on Provider Practice and Specialty (MD-PPAS) from 2016 through 2021, which includes all physicians who billed Medicare and are registered in the Provider, Enrollment, Chain and Ownership System. MD-PPAS data were only available through 2021; thus, acquisitions in 2022 were matched to 2021 MD-PPAS. NPIs were tracked over time for each practice. To focus on primary care, only practices where at least half of physicians had specialties of general practice (physician specialty 01), family medicine (physician specialty 08), internal medicine (physician specialty 11), or geriatric medicine (physician specialty 38) were included.

### Medicare Claims Data

Using 20% Medicare Part B claims, we identified all claims billed by PCPs defined as physicians with specialties listed above in each year from 2016 to 2022. We then assigned each patient to 1 PCP in a year using a previously established method^4^: in descending order of priority, highest number of Evaluation and Management visits, higher Medicare spending determined by allowable charge, and follow-up annual wellness visit. This left 1.67% of more than 55 million patient-NPI combinations unassigned, which were subsequently dropped.

### Study Outcomes

Primary outcomes at the patient-quarter level were number of hospitalizations (defined as distinct admission dates in MedPAR including observation stays) and number of emergency department (ED) visits (distinct visit dates in Part B claims data with place of service code 23). We further classified each hospitalization as a potentially avoidable hospitalization for ambulatory care–sensitive conditions (ACSCs) using *International Statistical Classification of Diseases and Related Health Problems, Tenth Revision* codes defined by Agency for Healthcare Research and Quality prevention quality indicators PQI 90 (overall composite indicator) and PQI 92 (chronic composite indicator). Secondary outcomes examined potential changes in patient composition using data from the Medicare Master Beneficiary Summary File, including age, sex, proportion of patients who were White, and mean Hierarchical Condition Category (HCC) score. We included race and ethnicity in the analysis to determine changes in patient composition along these dimensions, given well-documented disparities in care by race and ethnicity. The proportion of patients who were White was included as a measure to maximize sample size.

### Statistical Analysis

We used difference-in-differences (DID) regressions to measure the association between PE acquisition and outcomes of interest. We used a stacked difference-in-differences design to avoid bias that can occur with staggered treatment timing.^50,51,52,53^

Because practices acquired by PE may be systematically different than non-PE practices, the full universe of non-PE patients may not be an appropriate comparison group. We identified a comparison group of patients who were observably similar to acquired patients by matching each patient in an acquired practice with up to 5 similar patients in 2016, prior to any acquisition. Each patient in 2016 who was assigned to a physician that would eventually be acquired by PE was matched without replacement using a 1-SD match on age and HCC score and exact matching for sex, race and ethnicity, state of residence, and whether the patient had any dual eligibility (partial or full) with Medicaid for at least 1 month in 2016.

We compared preacquisition patient characteristics for patients with PE-acquired PCPs and matched controls by examining parallel trends in preacquisition group differences to detect any remaining imbalance after matching. In the event study analyses, event time 0 denoted the quarter of acquisition. We used data from 1.5 years before acquisition (event time −6, …, −1 quarter[s]) through 1.5 years after (event time +1, +2, …, +6 quarter[s]), with the quarter of acquisition as the reference period. We tested for differences in preacquisition trends between acquired practices and the control group by performing joint *F* tests of the hypothesis and that preacquisition interactions between the treatment and time indicators were no different (eTable 3 in Supplement 1).

The DID regressions for outcomes at the patient-quarter level included physician, patient, and time fixed effects. Thus, the difference-in-differences regressions estimate the within-physician and within-beneficiary change in outcomes following acquisition. The inclusion of fixed effects accounts for time-invariant attributes of physicians (eg, sex) and beneficiaries (eg, baseline presence of chronic conditions) that are associated with outcomes of interest. Therefore, we did not include further covariates in our regression model to account for additional fixed characteristics of physicians or beneficiaries. Standard errors were clustered according to each physician. Additional details regarding the statistical analysis are provided in the eMethods in Supplement 1. A 2-sided α of .05 was considered statistically significant. Analyses were conducted between November 2024 and February 2026 using Stata, version 18.0 (StataCorp LLC).

### Sensitivity Analysis

In sensitivity analysis, we examined outcomes defined as binary variables (probabilities) rather than count variables, ie, the probabilities of hospitalization, overall preventable hospitalization, and potentially chronic preventable hospitalization, and ED visits (eTable 4 in Supplement 1). Given that the sets of outcomes we examined were often binary or occurred with a low frequency, the linear regression models we used in our DID analyses may not be appropriate. In sensitivity analyses, we used Poisson/logit specifications with the Woolridge DID estimator^54^ to test the robustness of certain rare outcomes (eTable 5 in Supplement 1).

We also tested each outcome without patient fixed effects (eTable 6 in Supplement 1), with a natural log of outcomes (eTable 7 in Supplement 1), with an alternative matching strategy using just state and HCC score (eTable 8 in Supplement 1), using all potential controls rather than a matched sample (eTable 9 in Supplement 1), and only for acquisitions from before 2020 to test longer post-periods (eTable 10 in Supplement 1). We examined the robustness of our results using the Callway and Sant’Anna estimator (eTable 11 in Supplement 1).^50^ Given the potential for workforce turnover at PE-acquired practices,^28,29^ we also examined the robustness of our results to the exclusion of physician fixed effects (eTable 12 in Supplement 1).

Given that our patient-PCP attribution algorithm required patients to be attributed to a PCP every year, we next addressed potential concerns from this approach through several additional tests. We examined the robustness of our results to the inclusion of interaction terms for beneficiary and physician fixed effects to estimate the change in outcomes within each beneficiary and physician pair (eTable 13 in Supplement 1). Finally, we conducted a sensitivity analysis that limits the sample to those patients that were attributed to the same PCP for all 7 years in the data. This restriction eliminated potential bias due to patient selection in or out of a PCP’s panel following acquisition. Results are presented in eTable 14 in Supplement 1. The various analyses for each outcome are summarized in eTable 15 in Supplement 1.

## Results

The final sample included 24 397 Medicare beneficiaries with PE-acquired PCPs matched to 121 939 control Medicare beneficiaries, with 24 380 (99.9%) matching to a full set of 5 controls. Participants’ mean (SD) age of 74 (10) years. Overall, 56% of patients were female and 44% were male; 8.4% had any dual eligibility with Medicaid; and the mean (SD) HCC score was 1.27 (1.24) in treated patients and 1.29 (1.26) in control patients. At baseline, the racial and ethnic composition of the sample was 1.2% Asian, 5.8% Black, 4.8% Hispanic, 86.4% White, 0.6% other, and 1.2% unknown. The mean (SD) proportions of all patient characteristics and the standardized mean differences between PE and control patients are shown in Table 1. Counts of active PE affiliation by year are shown in eTable 1 in Supplement 1; additional details on matching and covariate balance are reported in Table 1 and eTable 2 in Supplement 1.

**Table 1.** Characteristics of Patients With Private Equity (PE)–Acquired Primary Care Physicians and Matched Non-PE Controls, 2016^a^

Characteristic	Proportion, mean (SD)^b^		% SMD
	Non-PE (n = 121 939)	PE (n = 24 397)	
Age, y	74 (10)	74 (10)	−0.4
HCC score, points	1.29 (1.26)	1.27 (1.24)	−0.7
Sex			
Female	0.56 (0.50)	0.56 (0.50)	0.0
Male	0.44 (0.50)	0.44 (0.50)	0.0
Dual eligibility	0.08 (0.28)	0.08 (0.28)	0.1
Race and ethnicity			
American Indian or Alaska Native	0.001 (0.03)	0.001 (0.03)	0.3
Asian	0.012 (0.11)	0.012 (0.11)	0.0
Black	0.058 (0.23)	0.058 (0.23)	0.0
Hispanic	0.048 (0.21)	0.048 (0.21)	0.0
White	0.863 (0.34)	0.863 (0.34)	−0.1
Other^c^	0.006 (0.08)	0.006 (0.08)	0.1
Unknown	0.012 (0.11)	0.012 (0.11)	0.1

Abbreviations: HCC, Hierarchical Condition Category; SMD, standardized mean difference.

^a^ Patients were matched using a 1-SD caliper with age and HCC score and exact match on sex, race, state of residence, and dual eligibility. The dual-eligibility variable equals 1 when the beneficiary had at least 1 month of partial or full dual eligibility with Medicaid in 2016. Four patients who saw PE-acquired physicians went unmatched. % SMD represents the standardized mean difference multiplied by 100.

^b^ Unless otherwise indicated.

^c^ Cannot be broken down further.

### Patient Outcomes

**Figure 1.**
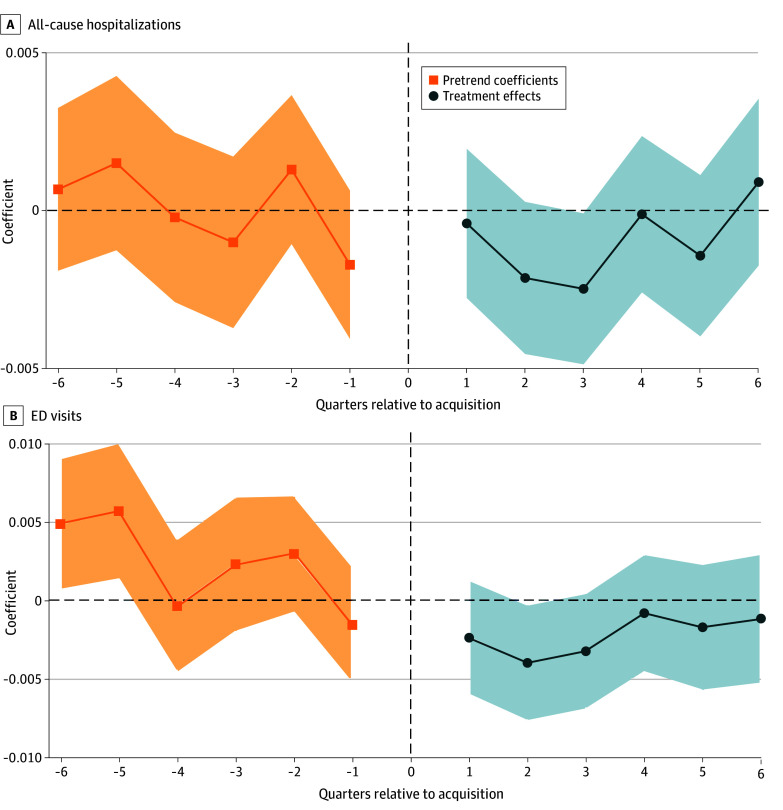
Differential Change in Number of All-Cause Hospitalizations and Emergency Department (ED) Visits, Private Equity (PE) and Matched Controls, 2016 to 2022 Each point represents the coefficient obtained by estimating a stacked difference-in-differences event study regression that compared patients assigned to PE-acquired physicians with patients in matched controls, before and after acquisition. The vertical dashed line represents the quarter of acquisition. The unit of analysis was the patient-quarter level. Event study regressions include patient, physician, and time fixed effects. Standard errors, represented by shaded areas, are clustered at the physician level.

After PE acquisition, the number of all-cause ED visits decreased by 0.002 (95% CI, −0.004 to −0.0002) visits per patient-quarter, or −1.36% (95% CI, −2.72% to −0.14%) relative to baseline (Table 2; Figure 1). The number of all-cause hospitalizations also declined by 0.001 (95% CI, −0.002 to 0.0003) admissions per patient-quarter reduction, or −1.49% (95% CI, −2.99% to 0.45%) after PE acquisition, but this decrease was not significant at α = .05 (Table 2; Figure 1). There was no significance change in the number of potentially preventable admissions (both PQI 90 and PQI 92) (eFigure in Supplement 1).

**Table 2.** Differential Change in Outcomes of Interest, Private Equity (PE) and Matched Controls, 2016 to 2022^a^

Outcome	Preacquisition visits per patient-quarter, mean (SD)	DID, No. per patient-quarter (95% CI)	DID % (95% CI)	*P* value
Hospitalizations				
No. of all-cause admissions	0.067 (0.315)	−0.001 (−0.002 to 0.0003)	−1.49 (−2.99 to 0.45)	.15
No. of potentially preventable admissions	0.010 (0.110)	0.00002 (−0.0004 to 0.0005)	0.20 (−4.00 to 5.00)	.92
No. of potentially preventable admissions for chronic conditions	0.007 (0.089)	−0.0001 (−0.0004 to 0.0003)	−1.43 (−5.71 to 4.29)	.70
ED use				
No. of all-cause ED visits	0.147 (0.597)	−0.002 (−0.004 to −0.0002)	−1.36 (−2.72 to −0.14)	.03

Abbreviations: DID, difference-in-differences; ED, emergency department.

^a^ DID regression coefficients are estimated using a stacked DID model that includes patient, physician, and time fixed effects. Standard errors are clustered at the level of the assigned primary care physician. Adjusted percentage differential change is calculated by dividing the adjusted differential change obtained from the DID regression, by the preacquisition mean for PE-acquired physicians.

### Patient Composition

**Figure 2.**
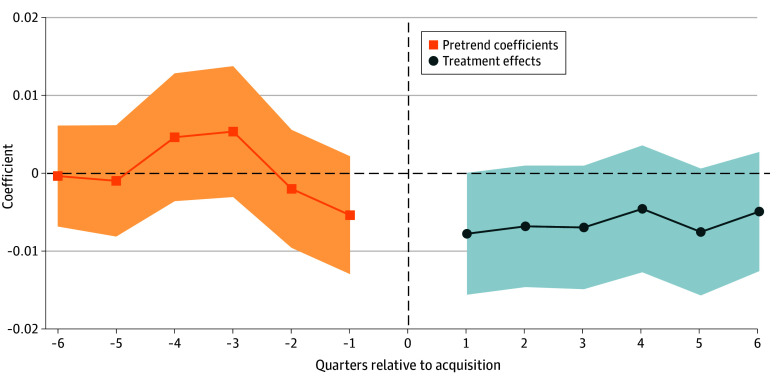
Differential Change in Hierarchical Condition Category Score, Private Equity (PE) and Matched Controls, 2016 to 2022 Each point represents the coefficient obtained by estimating a stacked difference-in-differences event study regression that compared patients assigned to PE-acquired physicians with patients in matched controls, before and after acquisition. The vertical dashed line represents the quarter of acquisition. The unit of analysis was the patient-quarter level. Event study regressions include patient, physician, and time fixed effects. Standard errors, represented by shaded areas, are clustered at the physician level.

In terms of the patient population seen by PE vs non-PE PCPs, differential changes in age, proportion of White patients, and proportion of female patients were not significant (Table 3). Furthermore, though the mean HCC score of patients seen by PE physicians decreased by 0.0064 units (95% CI, −0.013 to 0.0004 units) or −0.50% (95% CI, −1.02% to 0.03%) relative to the preacquisition mean, the decrease was not statistically significant at α = .05 (Table 3; Figure 2).

**Table 3.** Differential Change in Patient Composition, Private Equity (PE) and Matched Controls, 2016 to 2022^a^

Variable	Preacquisition mean (SD)	*F* test statistic	*P* value for *F* test	DID (95% CI)	DID %	*P* value for DID
Age	73.7 (9.8)	1.52	.18	0.0001 (−0.0003 to 0.0005)	0.0001 (−0.0004 to 0.0007)	.53
Proportion female	0.557 (0.50)	1.84	.10	0.0012 (−0.0007 to 0.0032)	0.22 (−0.13 to 0.57)	.20
Proportion White	0.863 (0.34)	3.17	.01	0.00017 (−0.0010 to 0.0013)	0.02 (−0.12 to 0.15)	.78
HCC score	1.27 (1.24)	3.04	.01	−0.0064 (−0.013 to 0.0004)	−0.50 (−1.02 to 0.03)	.07

Abbreviations: HCC, Hierarchical Condition Category; DID, difference-in-differences.

^a^ Unadjusted and adjusted differential changes in outcome variables are averaged at the patient-quarter level for PE patients and matched controls. Adjusted regression coefficients are estimated using a stacked DID model that includes physician, and time fixed effects for proportion female and proportion White and beneficiary, physician, and time fixed effects for age and HCC score. Standard errors are clustered at the level of the assigned primary care physician. Adjusted percentage differential change is calculated by dividing the adjusted differential change obtained from the DID regression, by the preacquisition mean for PE-acquired physicians.

### Sensitivity Analyses

Joint *F* tests to assess differences in preacquisition trends showed no differences in outcomes of interest between patients with PE-acquired PCPs and matched controls (eTable 3 in Supplement 1). There were no significant changes in the probabilities of all-cause hospitalizations, potentially preventable hospitalizations, preventable hospitalizations for chronic conditions, and all-cause ED visits per patient-quarter relative to baseline (eTable 4 in Supplement 1).

The Woolridge DID with logit (binary outcomes) or Poisson (count outcomes) specifications was used as a robustness check due to the outcomes being relatively rare. This statistical analysis found a statistically significant reduction in the number of ED visits and also found the probability of all-cause hospitalizations and ED visits to decrease by 0.38% (95% CI, −0.61% to −0.16%) and 0.75% (95% CI, −1.11% to −0.40%), respectively (eTable 5 in Supplement 1).

DID analysis without beneficiary fixed effects (eTable 6 in Supplement 1) found statistically significant increases in the number of potentially preventable hospitalizations; however, this specification also found simultaneous increase in the mean age and HCC score of patients at PE-acquired practices vs matched controls. There were no statistically significant differences between PE patients and matched controls when using natural log-transformed outcomes (eTable 7 in Supplement 1).

An alternative matching strategy that relied on state of residence and baseline HCC score found statistically significant reductions in the number and probability of all-cause ED visits (eTable 8 in Supplement 1), and the sensitivity test using all controls found statistically significant decreases in the number and probability of potentially preventable hospitalizations (eTable 9 in Supplement 1). Sensitivity analyses limited to acquisitions prior to 2020 and using the Callaway and Sant’Anna estimator found no significant changes in outcomes (eTables 10 and 11 in Supplement 1). The statistically significant reduction in the number of ED visits was robust to removal of physician fixed effects (eTable 12 in Supplement 1), inclusion of fixed effects for each beneficiary-physician pair (eTable 13 in Supplement 1), and limiting the sample to consistent beneficiary-physician pairings for the entire duration of the study period (eTable 14 in Supplement 1).

## Discussion

PE has recently expanded into health care by acquiring physician practices, with uncertain and unknown impacts on care quality. In this national study of traditional Medicare beneficiaries, we found that PE acquisitions of primary care practices from 2016 to 2022 were not associated with meaningful short-term changes in acute care outcomes. Patients seeing PE-acquired physicians experienced a small reduction in emergency department visits (1.36%), though this finding was not robust across all model specifications. We found no statistically significant changes in potentially preventable hospitalizations. Additionally, PE acquisitions did not appear to shift patient composition toward healthier populations as measured by observable patient characteristics, including HCC scores.

Our findings require careful interpretation in light of several methodological considerations. First, potentially preventable hospitalizations and emergency department visits may require longer follow-up periods to detect meaningful changes. Second, while these outcomes are not rare in absolute terms (affecting millions of Medicare beneficiaries annually), the base rates within individual practices may limit statistical power, particularly given PE’s recent entry into primary care. Third, our finding that patients with PE-acquired PCPs experienced a marginal reduction in emergency department visits merits particular attention. Although this finding was directionally consistent across all specifications, results were not significant on incorporating different follow-up periods and without patient fixed effects. This variation likely reflects several factors, including potential differences in unobserved patient composition between PE-acquired and control practices and heterogeneity in PE firm strategies and implementation timelines. Given the variation across model specifications and the modest reductions in ED visits, we interpret our findings as suggestive of minimal short-term impact rather than definitive evidence of patient benefit.

Our findings contribute to a growing literature on PE investments and patient outcomes. While prior studies have documented adverse outcomes in nursing homes and hospitals,^18,19,20,21,22,23,24^ our results suggest that PE’s effects on care quality are not uniform across sectors. Within primary care, our findings suggest that PE ownership does not immediately degrade core primary care functions. However, important caveats remain. First, our short follow-up period may precede the manifestation of harm if quality erosion emerges as firms pursue returns later in the ownership cycle. Second, PE investments may alter other dimensions, including patient experience, care coordination, and clinician burnout, that our outcomes do not capture.

More broadly, our findings should not be interpreted as evidence that PE acquisitions of physician practices should proceed without oversight. Previous research has identified specific concerns emerging from PE’s “platform and add-on” model of consolidation that has been shown to increase health care prices without improving quality or patient care.^10,13,14,22,25,55^ Within primary care, our prior work found PE-acquired primary care practices negotiate 8% higher commercial prices relative to independent practices.^1^ Given that PE investments do not drive measurable improvements or immediate harm to outcomes in the short term, future research must examine whether this cost-quality mismatch persists or worsens over longer time horizons. Key priorities for policy include mandatory disclosure requirements for acquisitions to promote ownership transparency and facilitate timely detection of patient concerns, as well as greater antitrust enforcement of PE’s platform and add-on model of consolidation that can alter the competitive landscape of primary care through incremental acquisitions that drive higher prices and workforce disruptions.^1,7,27,28,55,56^

### Limitations

This study has limitations. First, given the lack of systematic reporting and disclosure requirements, it is likely we underestimated PE’s true penetration into primary care.^57^ Second, because many PE acquisitions in primary care occurred during and after 2020, our findings should be interpreted as the short-term association of PE acquisitions with patient outcomes. Although we conducted a sensitivity analysis examining a longer follow-up period for acquisitions prior to 2020, this analysis is likely to be underpowered to detect any meaningful differences in patient outcomes. Third, though patients in acquired and nonacquired practices may be similar, the practices where their PCPs work may differ in unobserved ways that impact our outcomes. Fourth, we were not able to separately examine care provided by advanced practitioners, who make up a growing part of the primary care workforce and bill under physician NPIs; this omission may introduce measurement error to our patient-PCP attribution algorithm. Moreover, previous studies have documented changes to workforce composition and turnover following PE acquisitions^27,28,29^; whether PE acquisitions in primary care are accompanied by similar workforce shifts warrants further study. Fifth, our matched controls may include patients who see physicians from independent practices, hospital-affiliated practices, and payer-affiliated practices, which may not be a homogenous comparison group. Relatedly, use of fee-for-service Medicare data may not generalize to other patient populations or sources of insurance coverage, including the Medicare Advantage population. Sixth, while we both adjust for patient time-varying observed characteristics and use patient-level fixed effects, other factors may contemporaneously influence quality outcomes following PE acquisition.

## Conclusions

In this differences-in-differences study, PE acquisitions in primary care did not yield meaningful changes in all-cause or preventable hospitalizations or in ED visits for the traditional Medicare population. Findings contribute to ongoing policy discourse on the role of PE investments in shaping patient outcomes, suggesting heterogeneity in outcomes across health care settings.

## Notes

Supplement 1.
eMethods.
**eTable 1.** Active PE vs. non-PE patient and physician population by year, 2016-2022 **eTable 2.** Characteristics of PE-exposed patients and matched controls, 2016 **eTable 3.** Joint F tests **eTable 4.** Differential change in binary outcomes of interest, PE and matched controls, 2016-2022 **eTable 5.** Differential change in probabilities of outcomes of interest, PE and matched controls, 2016-2022 (Woolridge DID Logit/Poisson Specifications) **eTable 6.** Differential change in outcomes of interest without beneficiary fixed effects, PE and matched controls, 2016-2022 **eTable 7.** Differential change in natural log transformed outcomes of interest, PE and matched controls, 2016-2022 **eTable 8.** Differential change in outcomes of interest with alternative matching strategy, PE and matched controls, 2016-2022 **eTable 9.** Differential change in outcomes of interest with randomized acquisition dates for controls, PE and all controls, 2016-2022 **eTable 10.** Differential change in outcomes of interest, PE and matched controls with acquisition dates prior to 2020, 2016-2022 **eTable 11.** Differential change in outcomes of interest without physician fixed effects, PE and matched controls, 2016-2022 **eTable 12.** Differential change in outcomes of interest, PE and matched controls, 2016-2022 [Callway and Sant’Anna estimator] **eTable 13.** Differential change in outcomes of interest with patientXphysician fixed effects, PE and matched controls, 2016-2022 **eTable 14.** Differential change in outcomes of interest [balanced panel], PE and matched controls, 2016-2022 **eTable 15.** Summary of outcomes across specifications **eFigure.** Stacked event studies of outcomes of interest, PE and matched controls, 2016-2022

Supplement 2.
Data Sharing Statement

## References

[1] Singh Y, Radhakrishnan N, Adler L, Whaley C. Growth of private equity and hospital consolidation in primary care and price implications. JAMA Health Forum. 2025;6(1):e244935. doi:10.1001/jamahealthforum.2024.4935 39820388 PMC11742525

[2] Meyers DJ, Shroff J, Singh Y, Whaley CM. Corporate practice of medicine: vertical alignment and Medicare advantage risk coding. Center for Advancing Health Policy Through Research Digital Collection. Brown Digital Repository. Brown University Library. October 2025. Accessed March 27, 2026. 10.26300/amm6-vh76

[3] Adler L, Crow S, Fiedler M, . The changing landscape of primary care: an analysis of payer-primary care integration. Health Aff Sch. 2025;3(7):qxaf120. doi:10.1093/haschl/qxaf120 40612500 PMC12223493

[4] Whaley CM, Zhao X, Richards M, Damberg CL. Higher Medicare spending on imaging and lab services after primary care physician group vertical integration. Health Aff (Millwood). 2021;40(5):702-709. doi:10.1377/hlthaff.2020.01006 33939518 PMC9924392

[5] Ikram U, Aung KK, Song Z. Private equity and primary care: lessons from the field. Catal Non-Issue Content. 2021;2(6). doi:10.1056/CAT.21.0276

[6] Rooke-Ley H, Song Z, Zhu JM. Value-based payment and vanishing small independent practices. JAMA. 2024;332(11):871-872. doi:10.1001/jama.2024.12900 39172475 PMC12005269

[7] Singh Y, Reddy M, Zhu JM. Life cycle of private equity investments in physician practices: an overview of private equity exits. Health Aff Sch. 2024;2(4):qxae047. doi:10.1093/haschl/qxae047 38756171 PMC11044962

[8] FTC Challenges Private Equity Firm’s Scheme to Suppress Competition in Anesthesiology Practices Across Texas. Federal Trade Commission. 2023. Accessed May 13, 2024. https://www.ftc.gov/news-events/news/press-releases/2023/09/ftc-challenges-private-equity-firms-scheme-suppress-competition-anesthesiology-practices-across

[9] Singh Y, Song Z, Polsky D, Bruch JD, Zhu JM. Association of private equity acquisition of physician practices with changes in health care spending and utilization. JAMA Health Forum. 2022;3(9):e222886. doi:10.1001/jamahealthforum.2022.2886 36218927 PMC9440392

[10] Braun RT, Bond AM, Qian Y, Zhang M, Casalino LP. Private equity in dermatology: effect on price, utilization, and spending. Health Aff (Millwood). 2021;40(5):727-735. doi:10.1377/hlthaff.2020.02062 33939519

[11] La Forgia A, Bond AM, Braun RT, . Association of physician management companies and private equity investment with commercial health care prices paid to anesthesia practitioners. JAMA Intern Med. 2022;182(4):396-404. doi:10.1001/jamainternmed.2022.0004 35226052 PMC8886444

[12] Philips AP, Radhakrishnan N, Whaley CM, Singh Y. Hospital- and private equity-affiliated specialty physicians negotiate higher prices than independent physicians. Health Aff (Millwood). 2025;44(10):1226-1234. doi:10.1377/hlthaff.2025.00493 41052392

[13] Singh Y, Aderman CM, Song Z, Polsky D, Zhu JM. Increases in Medicare spending and use after private equity acquisition of retina practices. Ophthalmology. 2024;131(2):150-158. doi:10.1016/j.ophtha.2023.07.031 37557920 PMC12834666

[14] Braun RT, Lelli GJ, Pandey A, Zhang M, Winebrake JP, Casalino LP. Association of private equity firm acquisition of ophthalmology practices with Medicare spending and use of ophthalmology services. Ophthalmology. 2024;131(3):360-369. doi:10.1016/j.ophtha.2023.09.029 37777118 PMC10922192

[15] Singh Y, Cardenas GB, Torabzadeh H, Whaley CM, Borkar D. Private equity-owned physician practices decreased access to retinal detachment surgery, 2014-22. Health Aff (Millwood). 2025;44(5):589-596. doi:10.1377/hlthaff.2024.01204 40267368

[16] Arnold DR, Fulton BR, Abdelhadi OA, Teotia A, Scheffler RM. Private equity acquisition of gastroenterology practices and colonoscopy price and quality. JAMA Health Forum. 2025;6(6):e251476. doi:10.1001/jamahealthforum.2025.1476 40540284 PMC12181784

[17] La Forgia A, Bodner J. Getting down to business: chain ownership and fertility clinic performance. Management Sci. 2024;71(6):5022-5044. doi:10.1287/mnsc.2023.02793

[18] Braun RT, Yun H, Casalino LP, . Comparative performance of private equity–owned US nursing homes during the COVID-19 pandemic. JAMA Netw Open. 2020;3(10):e2026702. doi:10.1001/jamanetworkopen.2020.26702 33112402 PMC7593807

[19] Gupta A, Howell ST, Yannelis C, Gupta A. Owner incentives and performance in healthcare: private equity investment in nursing homes. Rev Financial Studies. 2024;37(4):1029-1077. doi:10.1093/rfs/hhad082

[20] Gandhi A, Song Y, Upadrashta P. Private equity, consumers, and competition: evidence from the nursing home industry. National Bureau of Economic Research. October 2025. Revised February 2026. Accessed February 5, 2026. https://www.nber.org/papers/w34306

[21] Kannan S, Bruch JD, Zubizarreta JR, Stevens J, Song Z. Hospital staffing and patient outcomes after private equity acquisition. Ann Intern Med. 2025;178(11):1529-1538. doi:10.7326/ANNALS-24-03471 40982974

[22] Kannan S, Bruch JD, Song Z. Changes in hospital adverse events and patient outcomes associated with private equity acquisition. JAMA. 2023;330(24):2365-2375. doi:10.1001/jama.2023.23147 38147093 PMC10751598

[23] Bhatla A, Bartlett VL, Liu M, Zheng Z, Wadhera RK. Changes in patient care experience after private equity acquisition of US hospitals. JAMA. 2025;333(6):490-497. doi:10.1001/jama.2024.23450 39786740 PMC11815525

[24] Diaz A, Mead M, Rohde S, Kunnath N, Dimick JB, Ibrahim AM. Hospitals acquired by private equity firms: increased postoperative mortality for common inpatient surgeries. Health Aff (Millwood). 2025;44(5):554-562. doi:10.1377/hlthaff.2024.01102 40324137

[25] Borsa A, Bejarano G, Ellen M, Bruch JD. Evaluating trends in private equity ownership and impacts on health outcomes, costs, and quality: systematic review. BMJ. 2023;382:e075244. doi:10.1136/bmj-2023-075244 37468157 PMC10354830

[26] La Forgia A, McDevitt RC. How should we assess quality of health care services in organizations owned by private equity firms? AMA J Ethics. 2025;27(5):E385-E391. doi:10.1001/amajethics.2025.385 40315115

[27] Berquist V, Klarnet L, Dafny L. Sale of private equity-owned physician practices and physician turnover. JAMA Health Forum. 2025;6(2):e245376. doi:10.1001/jamahealthforum.2024.5376 39951313 PMC11829224

[28] Singh Y, Cardenas GB, Torabzadeh H, Borkar D, Whaley CM. Physician turnover increased in private equity-acquired physician practices. Health Aff (Millwood). 2025;44(3):280-287. doi:10.1377/hlthaff.2024.00974 40030104

[29] Bruch JD, Foot C, Singh Y, Song Z, Polsky D, Zhu JM. Workforce composition in private equity-acquired versus non-private equity-acquired physician practices. Health Aff (Millwood). 2023;42(1):121-129. doi:10.1377/hlthaff.2022.00308 36623222

[30] Singh Y, Cantor J, Whaley CM, Shuey B, Bilden R, Donahoe JT. Private equity acquiring large shares of the opioid treatment market without changing market-level methadone supply. Health Aff (Millwood). 2025;44(9):1181-1189. doi:10.1377/hlthaff.2025.00326 40893070

[31] Jiao YA. The impact of private equity hospital acquisitions on maternal health for Medicaid patients. Health Serv Res. Published online October 4, 2025. doi:10.1111/1475-6773.70048 PMC12857500 41045029

[32] Richards MR, Whaley CM. Hospital behavior over the private equity life cycle. J Health Econ. 2024;97:102902. doi:10.1016/j.jhealeco.2024.102902 38861907 PMC11392649

[33] Bindman AB, Grumbach K, Osmond D, . Preventable hospitalizations and access to health care. JAMA. 1995;274(4):305-311. doi:10.1001/jama.1995.03530040033037 7609259

[34] Booker M, Purdy S. Towards new definitions of avoidable hospital admissions. Br J Gen Pract. 2022;72(723):464-465. doi:10.3399/bjgp22X720725

[35] van Loenen T, van den Berg MJ, Westert GP, Faber MJ. Organizational aspects of primary care related to avoidable hospitalization: a systematic review. Fam Pract. 2014;31(5):502-516. doi:10.1093/fampra/cmu053 25216664

[36] Oh NL, Potter AJ, Sabik LM, Trivedi AN, Wolinsky F, Wright B. The association between primary care use and potentially-preventable hospitalization among dual eligibles age 65 and over. BMC Health Serv Res. 2022;22(1):927. doi:10.1186/s12913-022-08326-2 35854303 PMC9295296

[37] Fleming ST. Primary care, avoidable hospitalization, and outcomes of care: a literature review and methodological approach. Med Care Res Rev. 1995;52(1):88-108. doi:10.1177/107755879505200106 10143578

[38] Tabak R, Somers K, Pozzi P, Riley C. Potentially avoidable hospitalizations among Medicare and dual eligible enrollees. BRG. 2023. Accessed April 25, 2025. https://www.thinkbrg.com/insights/publications/av-imrad/

[39] McDermott KW, Jiang HJ. Characteristics and costs of potentially preventable inpatient stays, 2017 #259. AHRQ. 2020. Accessed April 25, 2025. https://hcup-us.ahrq.gov/reports/statbriefs/sb259-Potentially-Preventable-Hospitalizations-2017.jsp?utm_source=chatgpt.com 32730017

[40] Timmins L, Peikes D, McCall N. Pathways to reduced emergency department and urgent care center use: Lessons from the comprehensive primary care initiative. Health Serv Res. 2020;55(6):1003-1012. doi:10.1111/1475-6773.13579 33258126 PMC7704466

[41] Rosano A, Loha CA, Falvo R, . The relationship between avoidable hospitalization and accessibility to primary care: a systematic review. Eur J Public Health. 2013;23(3):356-360. doi:10.1093/eurpub/cks053 22645236

[42] Kozak LJ, Hall MJ, Owings MF. Trends in avoidable hospitalizations, 1980-1998. Health Aff (Millwood). 2001;20(2):225-232. doi:10.1377/hlthaff.20.2.225 11260947

[43] McCall N, Harlow J, Dayhoff D. Rates of hospitalization for ambulatory care sensitive conditions in the Medicare+Choice population. Health Care Financ Rev. 2001;22(3):127-145. 25372877 PMC4194704

[44] McCall N, Brody E, Mobley L, Subramanian S. Investigation of increasing rates of hospitalization for ambulatory care sensitive conditions among Medicare fee-for-service beneficiaries, final report. RTI International. 2004. https://www.cms.hhs.gov/Reports/Downloads/McCall_2004_3.pdf

[45] Torio CM, Andrews RM. Geographic Variation in Potentially Preventable Hospitalizations for Acute and Chronic Conditions, 2005–2011. In: Healthcare Cost and Utilization Project (HCUP) Statistical Briefs. Agency for Healthcare Research and Quality. 2006. 25411684

[46] Pappas G, Hadden WC, Kozak LJ, Fisher GF. Potentially avoidable hospitalizations: inequalities in rates between US socioeconomic groups. Am J Public Health. 1997;87(5):811-816. doi:10.2105/AJPH.87.5.811 9184511 PMC1381055

[47] Blustein J, Hanson K, Shea S. Preventable hospitalizations and socioeconomic status. Health Aff (Millwood). 1998;17(2):177-189. doi:10.1377/hlthaff.17.2.177 9558796

[48] Weissman JS, Gatsonis C, Epstein AM. Rates of avoidable hospitalization by insurance status in Massachusetts and Maryland. JAMA. 1992;268(17):2388-2394. doi:10.1001/jama.1992.03490170060026 1404795

[49] Abdelhadi O, Fulton BD, Alexander L, Scheffler RM. Private equity-acquired physician practices and market penetration increased substantially, 2012-21. Health Aff (Millwood). 2024;43(3):354-362. doi:10.1377/hlthaff.2023.00152 38437602

[50] Callaway B, Sant’Anna PHC. Difference-in-differences with multiple time periods. J Econom. 2021;225(2):200-230. doi:10.1016/j.jeconom.2020.12.001

[51] Goodman-Bacon A. Difference-in-differences with variation in treatment timing. National Bureau of Economic Research. September 2018. Accessed April 1, 2026. https://www.nber.org/papers/w25018

[52] Sun L, Abraham S. Estimating dynamic treatment effects in event studies with heterogeneous treatment effects. J Econometrics. 2021;225(2):175-199. doi:10.1016/j.jeconom.2020.09.006

[53] Wing C, Freedman SM, Hollingsworth A. Stacked difference-in-differences. National Bureau of Economic Research. January 2024. Accessed April 1, 2026. https://www.nber.org/papers/w32054

[54] Wooldridge JM. Two-way fixed effects, the two-way Mundlak regression, and difference-in-differences estimators. Social Science Research Network. Preprint posted online August 17, 2021. Accessed February 5, 2026. doi:10.2139/ssrn.3906345

[55] Singh Y, Whaley C. Private equity rollups of physician practices. Presented at ASHEcon 2025 Conference; June 23, 2025. Accessed February 10, 2026. https://ashecon.confex.com/ashecon/2025/meetingapp.cgi/Home/0

[56] Asil A, Ramos P, Starc A, Wollmann TG. Painful bargaining: evidence from anesthesia rollups. National Bureau of Economic Research. Accessed April 1, 2026. https://www.nber.org/papers/w33217

[57] Singh Y, Brown ECF. The missing piece in health care transparency: ownership transparency. Health Aff Forefr. September 22, 2023. Accessed February 5, 2026. doi:10.1377/forefront.20230921.886842

