# Supplementary material for: Private Equity Acquisition in Primary Care and Avoidable Hospitalizations
Source: JAMA Health Forum. 2026 May 8;7(5):e261045. doi: 10.1001/jamahealthforum.2026.1045 (PMC13156794; doi:10.1001/jamahealthforum.2026.1045)
Supplement: Supplement 1. — eMethods. eTable 1. Active PE vs. non-PE patient and physician population by year, 2016-2022 eTable 2. Characteristics of PE-exposed patients and matched controls, 2016 eTable 3. Joint F tests eTable 4. Differential change in binary outcomes of interest, PE and matched controls, 2016-2022 eTable 5. Differential change in probabilities of outcomes of interest, PE and matched controls, 2016-2022 (Woolridge DID Logit/Poisson Specifications) eTable 6. Differential change in outcomes of interest without beneficiary fixed effects, PE and matched controls, 2016-2022 eTable 7. Differential change in natural log transformed outcomes of interest, PE and matched controls, 2016-2022 eTable 8. Differential change in outcomes of interest with alternative matching strategy, PE and matched controls, 2016-2022 eTable 9. Differential change in outcomes of interest with randomized acquisition dates for controls, PE and all controls, 2016-2022 eTable 10. Differential change in outcomes of interest, PE and matched controls with acquisition dates prior to 2020, 2016-2022 eTable 11. Differential change in outcomes of interest without physician fixed effects, PE and matched controls, 2016-2022 eTable 12. Differential change in outcomes of interest, PE and matched controls, 2016-2022 [Callway and Sant’Anna estimator] eTable 13. Differential change in outcomes of interest with patientXphysician fixed effects, PE and matched controls, 2016-2022 eTable 14. Differential change in outcomes of interest [balanced panel], PE and matched controls, 2016-2022 eTable 15. Summary of outcomes across specifications eFigure. Stacked event studies of outcomes of interest, PE and matched controls, 2016-2022 [file jamahealthforum-e261045-s001.pdf]

# Supplemental Online Content

Dixit MN, Philips AP, Trivedi AN, Whaley C, Singh Y. Private equity acquisition in primary care and avoidable hospitalizations. *JAMA Health Forum*. 2026;7(5):e261045. doi:10.1001/jamahealthforum.2026.1045

**eMethods.**
**eTable 1.** Active PE vs. non-PE patient and physician population by year, 2016-2022
**eTable 2.** Characteristics of PE-exposed patients and matched controls, 2016
**eTable 3.** Joint F tests
**eTable 4.** Differential change in binary outcomes of interest, PE and matched controls, 2016-2022
**eTable 5.** Differential change in probabilities of outcomes of interest, PE and matched controls, 2016-2022 (Woolridge DID Logit/Poisson Specifications)
**eTable 6.** Differential change in outcomes of interest without beneficiary fixed effects, PE and matched controls, 2016-2022
**eTable 7.** Differential change in natural log transformed outcomes of interest, PE and matched controls, 2016-2022
**eTable 8.** Differential change in outcomes of interest with alternative matching strategy, PE and matched controls, 2016-2022
**eTable 9.** Differential change in outcomes of interest with randomized acquisition dates for controls, PE and all controls, 2016-2022
**eTable 10.** Differential change in outcomes of interest, PE and matched controls with acquisition dates prior to 2020, 2016-2022
**eTable 11.** Differential change in outcomes of interest without physician fixed effects, PE and matched controls, 2016-2022
**eTable 12.** Differential change in outcomes of interest, PE and matched controls, 2016-2022 [Callway and Sant'Anna estimator]
**eTable 13.** Differential change in outcomes of interest with patientXphysician fixed effects, PE and matched controls, 2016-2022
**eTable 14.** Differential change in outcomes of interest [balanced panel], PE and matched controls, 2016-2022
**eTable 15.** Summary of outcomes across specifications
**eFigure**. Stacked event studies of outcomes of interest, PE and matched controls, 2016-2022

This supplemental material has been provided by the authors to give readers additional information about their work.

© 2026 Dixit MN et al. JAMA Health Forum.

**eMethods. Additional background on data and empirical approach**

We built our dataset in multiple steps: (1) identified PE acquisitions of primary care practices using Pitchbook data, (2) identified physicians associated with each practice using the Medicare Data on Provider Practice and Specialty (MD-PPAS), (3) assigned patients from the 20% Medicare Part B fee-for-service claims to one primary care physician in a year, and (4) linked patient assignment and acquisition data to outcomes of interest using Part B claims, enrollment file, and the Medicare Provider Analysis and Review (MedPAR) file.

***Identifying PE Acquisitions***

Our main source of data on private equity transactions is a proprietary list of leveraged buyouts by PE firms in the "Clinics and outpatient services" sector, compiled by Pitchbook Inc.–a financial database widely used in research on mergers and acquisitions, including studies focused on PE in health care. However, because no single data source captures every PE acquisition of physician practices, one limitation of this dataset is the potential underreporting of certain acquisitions. To address this gap, we supplemented the Pitchbook data with manual searches. Note that PE acquisitions of primary care practices prior to 2016 represented less than 0.5% of all primary care physicians and thus were excluded from this study.

***Identifying TINs and physician NPIs at acquired practices***

After identifying the acquired practices, we conducted web searchers to determine the physician owner or owners and their National Provider Identifiers (NPIs), following methods used in previous studies.[1–3] Our process began by locating the organizational NPI for each acquired practice using the CMS National Plan and Provider Enumeration System (NPPES), which also lists the "Authorized Official." This individual is typically a physician (MD/DO) and is defined as a general partner, chairman of the board, chief financial officer, chief executive officer, direct owner of 5% or more of the provider, or someone with comparable status and authority within the organization. Next, we identified the individual NPI for the authorized official associated with each acquired practice. To ensure the accuracy of this approach, we also consulted archived versions of physician practice website and NPPES records to confirm the identity of the authorized official at the time of acquisition.

We subsequently linked each acquisition to its Tax Identification Number (TIN) by matching the authorized official's or practice owner's NPI, as identified from the NPPES data, to the Medicare Data on Provider Practice and Specialty (MD-PPAS) for the corresponding year of acquisition. The MD-PPAS includes all registered providers in the United States who have billed Medicare at least once and are listed in the Provider, Enrollment, Chain and Ownership System (PECOS); it contains information such as the legal business name, geographic location, TIN, and provider NPI. We matched acquisitions and owner NPIs to MD-PPAS records by aligning the year of acquisition with the same year in the MD-PPAS data for each year from 2016 to 2021. MD-PPAS data was not available for 2022, thus 2022 acquisitions were matched to 2021 MD-PPAS. Using the MD-PPAS, we then identified all NPIs linked to the acquired TIN at the time of acquisition. Although there may be some measurement error in distinguishing practice owners

© 2026 Dixit MN et al. JAMA Health Forum.

from partners or associates, our approach is not affected by the exact identity of the physician owner, as we ultimately capture all physician NPIs affiliated with the acquired practice.

To ensure our focus on primary care, only practices where at least half of physicians had specialties General Practice (physician specialty 01), Family Medicine (physician specialty 08), Internal Medicine (physician specialty 11), or Geriatric Medicine (physician specialty 38) were included.

The 50% cutoff effectively separates primary care-focused practices from multispecialty groups while maximizing sample size. The 50% threshold was selected based on the distribution of PCP representation across practices (see Figure below). The figure summarizes the percentage of practices with a given share of PCPs, prior to implementing the 50% cutoff threshold. Non-PE practices show a bimodal distribution: the majority have either no PCPs (single or multispecialty groups) or are predominantly primary care. PE-acquired practices show similar clustering; with a small number of zero-PCP practices we attribute to measurement error in identifying PE deals. Increasing the threshold to 60% or 70% would exclude fewer than 10% of current sample practices. These practices (with share of PCPs between 50 and 70%) do not differ meaningfully on key characteristics such as number of unique beneficiaries or total practice size (not shown).

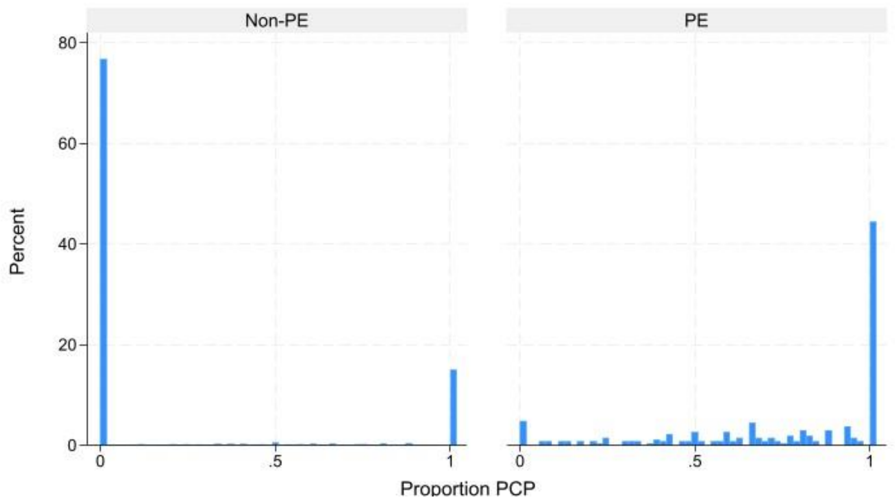


***Merge physicians via their NPI to Medicare fee-for-service claims data***
Within the 20% Medicare Part B claims, we gathered all "primary care" claims using the physician specialty code (codes 01, 08, 11, 38) and place of service code corresponding to the office setting (code 11). Following Whaley and Zhao (2024) we then assigned each patient to one primary care physician within a year by the amount of distinct Evaluation and Management claims.[4] Successive tiebreakers included the greater sum of Medicare allowable charge on claims billed within the year and whichever physician billed for the follow-up Medicare annual wellness visit. This method assigned all but 1.67% of more than 55 million patient-NPI combinations, which we excluded from the final analyses. Finally, we merged the PE acquisition database with the assigned patient-year-PCP dataset using physician NPIs. Patient characteristics and hospitalization outcomes were then obtained by merging the relevant data sources (MedPAR,

© 2026 Dixit MN et al. JAMA Health Forum.

Medicare Beneficiary Summary File) onto our patient-year-PCP dataset by the beneficiary identification number.

Our attribution algorithm requires patients to be attributed to a PCP every year. Thus, we allow patients to switch PCPs. To address the potential concerns from this approach, we conducted several additional tests including examining changes in the composition of beneficiaries attributed to a PCP following acquisition, additional analysis where we interact the beneficiary and provider fixed effects, and limiting the sample to beneficiaries attributed to the same PCP over the entire sample (i.e., only including patients in our sample that are attributed to the same PCP for all the 7 years in the data).

## **Empirical approach: matched difference-in-differences**

Our primary strategy relies on a differences-in-differences design. However, a concern when using this approach is that PE's decision to acquire certain practices (and thus its patients) is not exogenous, suggesting that acquired practices may be systematically different than non-PE practices. If we assume this to be the case, the full universe of non-PE patients may not be an appropriate control group.

### ***Constructing matched controls***

To address potential concerns regarding selection into treatment, we identify a control group of patients that are observably similar to PE-exposed patients at baseline. We construct a control group by matching each PE patient with up to 5 control patients, prior to any acquisition. The PE-acquired patients were matched to controls in 2016, the year prior to any acquisition in this study. Matched controls were identified using 5:1 matching without replacement and required matched controls to have the same sex, race/ethnicity, and relative exposure to dual-eligibility with Medicaid (binary variable which is equal to 1 if the beneficiary has any dual status for at least one month in the year) and within one standard deviation of mean age and HCC score. Overall, 25,397 PE-exposed patients were matched to 121,939 control patients, with 24,380 (99.9%) PE-acquired patients matched to a full set of 5 controls (see eTable 1 for counts of acquisitions by year and Table 1 for covariate balance).

### ***Stacked difference-in-differences***

We used the following regression to examine changes in outcomes of interest among PE-acquired patients to contemporaneous changes in outcomes of interest among matched controls:

$$y_{ijct} = \beta_0 + \beta_1 PE_{ijct} + \omega_i + \theta_j + \delta_t + \varepsilon_{ijct}$$

Where *i* indexes beneficiary, *c* indexes matched cohort, and *t* indexes time at the quarter-year level. $PE_{ict}$ identifies whether patient *i* in matched cohort *c* was seeing a PE-affiliated physician j in quarter-year *t*; $\omega_i$, $\theta_j$, and $\delta_t$, are beneficiary-, physician-, and time-fixed effects respectively, that control for time-invariant unobservable differences across physicians as well as secular time trends. With fixed effects, the difference-in-differences regression estimate the within-physician and within-beneficiary change in outcomes following acquisition thus we did not include

additional covariates in our regression model to account for additional fixed characteristics of physicians or beneficiaries.

Lastly, we used a stacked difference-in-differences design to compare outcomes for patients seeing PE-acquired primary care physicians with those of matched controls. We used a stacked DID rather than a traditional DID or two-way fixed-effects estimator to avoid bias that can occur with staggered treatment timing.[5–7]

### *Parallel Trends*

To test for parallel trends, we use an event study approach to estimate trends in the quarterly difference in outcomes between PE-exposed patients and matched controls. In the event study analyses, event time 0 denoted the quarter of acquisition. We used data from 1.5 years before acquisition (event time -6, …, -1 quarter[s]) through 1.5 years after (event time +1, +2, …, +6 quarter[s]), with the quarter of acquisition as the reference period.

## References


1. Braun RT, Bond AM, Qian Y, Zhang M, Casalino LP. Private Equity In Dermatology: Effect On Price, Utilization, And Spending. *Health Aff (Millwood)*. 2021;40(5):727-735. doi:10.1377/hlthaff.2020.02062

2. Braun RT, Lelli GJ, Pandey A, Zhang M, Winebrake JP, Casalino LP. Association of Private Equity Firm Acquisition of Ophthalmology Practices with Medicare Spending and Use of Ophthalmology Services. *Ophthalmology*. 2024;131(3):360-369. doi:10.1016/j.ophtha.2023.09.029

3. Singh Y, Cardenas GB, Torabzadeh H, Borkar D, Whaley CM. Physician Turnover Increased In Private Equity–Acquired Physician Practices. *Health Aff (Millwood)*. 2025;44(3):280-287. doi:10.1377/hlthaff.2024.00974

4. Whaley CM, Zhao X. The Effects of Physician Vertical Integration on Referral Patterns, Patient Welfare, and Market Dynamics. *J Public Econ*. 2024;238:105175. doi:10.1016/j.jpubeco.2024.105175

5. Callaway B, Sant'Anna PHC. Difference-in-Differences with multiple time periods. *J Econom*. 2021;225(2):200-230. doi:10.1016/j.jeconom.2020.12.001

6. Goodman-Bacon A. Difference-in-Differences with Variation in Treatment Timing. *National Bureau of Economic Research*. Preprint posted online September 2018:25018. doi:10.3386/w25018

7. Sun L, Abraham S. Estimating Dynamic Treatment Effects in Event Studies With Heterogeneous Treatment Effects. *Social Science Research Network*. Preprint posted online September 22, 2020:3158747. doi:10.2139/ssrn.3158747


© 2026 Dixit MN et al. JAMA Health Forum.

**eTable 1. Active PE vs. non-PE patient and physician population by year, 2016-2022**

| Year-Quarter | Private Equity Affiliated | | Not Private Equity | |
|---|---|---|---|---|
| | Patients | NPIs | Patients | NPIs |
| 2016 | 632 | 19 | 121,939 | 34,097 |
| 2017 | 841 | 41 | 101, 555 | 30,332 |
| 2018 | 978 | 50 | 91,758 | 28,720 |
| 2019 | 1,601 | 85 | 83,337 | 27,198 |
| 2020 | 3,359 | 197 | 72,352 | 24,855 |
| 2021 | 7,397 | 327 | 66,062 | 23,883 |
| 2022 | 6,850 | 350 | 59,538 | 22,621 |

Note: The table shows the number of distinct patients and physicians who were actively affiliated with private equity and the control population in a given year among the matched sample, based on 20% sample of Medicare fee for service data.

© 2026 Dixit MN et al. JAMA Health Forum.

**eTable 2. Characteristics of PE-exposed patients and matched controls, 2016**

| | Before Matching | | | After Matching | | |
|---|---|---|---|---|---|---|
| | Non-PE (n=4,041,348) | PE (n=24,401) | p-value | Non-PE (n=121,939) | PE (n=24,397) | p-value |
| Mean Age (SD) | 72.61 (11.07) | 73.70 (9.77) | <0.001 | 73.77 (9.85) | 73.70 (9.77) | 0.31 |
| Mean HCC score (SD) | 1.29 (1.32) | 1.27 (1.24) | 0.12 | 1.29 (1.26) | 1.27 (1.24) | 0.15 |
| Proportion (SD) | | | | | | |
| Female | 0.57 (0.50) | 0.56 (0.50) | <0.001 | 0.56 (0.50) | 0.56 (0.50) | 0.98 |
| Original Reason for Entitlement is Disability | 0.21 (0.40) | 0.15 (0.36) | <0.001 | 0.16 (0.36) | 0.15 (0.36) | 0.003 |
| ESRD | 0.008 (0.091) | 0.006 (0.080) | <0.001 | 0.006 (0.08) | 0.006 (0.08) | 0.60 |
| Any Dual-Eligibility | 0.16 (0.37) | 0.08 (0.28) | <0.001 | 0.08 (0.28) | 0.08 (0.28) | 0.93 |
| Race/Ethnicity, Proportion (SD) | | | | | | |
| Asian | 0.027 (0.16) | 0.012 (0.109) | <0.001 | 0.012 (0.11) | 0.012 (0.11) | 1.00 |
| Black | 0.082 (0.274) | 0.058 (0.234) | <0.001 | 0.058 (0.23) | 0.058 (0.23) | 1.00 |
| Hispanic | 0.050 (0.219) | 0.048 (0.213) | 0.037 | 0.048 (0.21) | 0.048 (0.21) | 1.00 |
| NA/AI | 0.003 (0.056) | 0.001 (0.033) | <0.001 | 0.001 (0.03) | 0.001 (0.03) | 0.71 |
| Other | 0.008 (0.087) | 0.006 (0.077) | 0.007 | 0.006 (0.08) | 0.006 (0.08) | 0.94 |
| Unknown | 0.013 (0.113) | 0.012 (0.109) | 0.15 | 0.012 (0.11) | 0.012 (0.11) | 0.89 |
| White | 0.82 (0.386) | 0.86 (0.344) | <0.001 | 0.86 (0.34) | 0.86(0.34) | 0.93 |

Note: Patients were matched using a 1 SD caliper with age and HCC score and exact match on sex, race, state of residence, and dual-eligibility. The dual-eligibility variable equals 1 when the beneficiary had at least one month of partial or full dual-eligibility with Medicaid in 2016. Four patients who saw PE-acquired physicians went unmatched.

© 2026 Dixit MN et al. JAMA Health Forum.

**eTable 3. Joint F tests**

| | F-Test Statistic | p-value |
|---|---|---|
| **Hospitalizations** | | |
| # of all-cause admissions | 1.22 (0.2960) | 0.30 |
| # of potentially preventable admissions | 0.87 (0.5002) | 0.50 |
| # of potentially preventable admissions for chronic conditions | 0.90 (0.4811) | 0.48 |
| **Emergency Department Use** | | |
| # of all-cause ED visits | 2.07 (0.0658) | 0.07 |

Note: Table reports results of joint F-Test of the hypothesis that pre-acquisition interactions between the treatment and time indicators were no different.

© 2026 Dixit MN et al. JAMA Health Forum.

## eTable 4. Differential change in binary outcomes of interest, PE and matched controls, 2016-2022

| | Pre-acquisition mean (SD) | DiD [95% C.I.] | DiD % | p-value |
|---|---|---|---|---|
| **Hospitalizations** | | | | |
| Any all-cause admissions | 0.0534082 (0.2248507) | -0.0004989 [-0.0013827, 0.000385] | -0.93 [-2.59, 0.72] | 0.269 |
| Any potentially preventable admissions | 0.0094274 (0.096638) | 0.000062 [-0.000315, 0.000439] | 0.66 [-3.34, 4.66] | 0.747 |
| Any potentially preventable admissions for chronic conditions | 0.0060663 (0.0776516) | -0.0000155 [-0.0003253, 0.0002943] | -0.26 [-5.36, 4.85] | 0.922 |
| **Emergency Department Use** | | | | |
| Any all-cause ED visits | 0.1034144 (0.3045055) | -0.0009755 [-0.0021154, 0.0001644] | -0.94 [-2.05, 0.16] | 0.093 |

Notes/Sources: Unadjusted and adjusted differential changes in outcome variables are averaged at the patient-quarter level for PE patients and matched controls. Adjusted regression coefficients are estimated using a stacked difference-in-differences model that includes **patient, physician, and time fixed effects**. Standard errors are clustered at the level of the assigned primary care physician. Adjusted percentage differential change is calculated by dividing the adjusted differential change obtained from the difference-in-differences regression, by the pre-acquisition mean for PE-acquired physicians.

© 2026 Dixit MN et al. JAMA Health Forum.

**eTable 5. Differential change in probabilities of outcomes of interest, PE and matched controls, 2016-2022 (Woolridge DID Logit/Poisson Specifications)**

| | Pre-acquisition mean (SD) | DiD [95% C.I.] | DiD % | p-value |
|---|---|---|---|---|
| **Hospitalizations** | | | | |
| Any all-cause admissions | 0.0534082 (0.2248507) | -0.0038448 [-0.006128, -0.0015616] | -0.38 [-0.61, -0.16] | **0.001** |
| # of all-cause admissions | 0.067 (0.315) | -0.0047071 [-0.0112422, 0.0018281] | -0.005 [-0.01, 0.002] | 0.158 |
| Any potentially preventable admissions | 0.0094274 (0.096638) | -0.0005701 [-0.0015929, 0.0004526] | -0.06 [-0.16, 0.05] | 0.275 |
| # of potentially preventable admissions | 0.010 (0.110) | -0.000257 [-0.0026563, 0.0021423] | -0.0003 [-0.003, 0.002] | 0.834 |
| Any potentially preventable admissions for chronic conditions | 0.0060663 (0.0776516) | -0.0004255 [-0.0012534, 0.0004025] | -0.04 [-0.13, 0.04] | 0.314 |
| # of potentially preventable admissions for chronic conditions | 0.007 (0.089) | -0.0002671 [-0.0021212, 0.001587] | -0.0003 [-0.002, 0.002] | 0.778 |
| **Emergency Department Use** | | | | |
| Any all-cause ED visits | 0.1034144 (0.3045055) | -0.0075136 [-0.0110556, -0.0039715] | -0.75 [-1.11, -0.40] | **<0.001** |
| # of all-cause ED visits | 0.147 (0.597) | -0.0117067 [-0.0218577, -0.0015557] | -0.012 [-0.02, -0.002] | **0.024** |

Notes/Sources: Unadjusted and adjusted differential changes in outcome variables are averaged at the patient-quarter level for PE patients and matched controls. Adjusted regression coefficients are estimated using a difference-in-differences model using the ETWFE estimator proposed by Woolridge and includes beneficiary, physician, and time fixed effects. Standard errors are clustered at the level of the assigned primary care physician. For binary outcomes, the logit method was specified, whereas the Poisson method was used for count outcomes. For binary probabilities, adjusted percentage differential change is calculated by multiplying the adjusted differential change obtained from the logit difference-in-differences regression by 100. For numeric variables, adjusted percentage differential change is calculated using $e^{\beta} - 1$ where $\beta$ is the adjusted differential change obtained from the Poisson difference-in-differences regression.

© 2026 Dixit MN et al. JAMA Health Forum.

**eTable 6. Differential change in outcomes of interest without beneficiary fixed effects, PE and matched controls, 2016-2022**

| | Pre-acquisition mean (SD) | DiD [95% C.I.] | DiD % | p-value |
|---|---|---|---|---|
| **Hospitalizations** | | | | |
| # of all-cause admissions | 0.0534082 (0.2248507) | 0.0010598 [-0.0003043, 0.0024238] | 1.98 [0.57, 4.54] | 0.128 |
| Any all-cause admission | 0.067 (0.315) | 0.0008938 [-0.000023, 0.0018105] | 1.33 [-0.03, 2.70] | 0.056 |
| # of potentially preventable admissions | 0.0094274 (0.096638) | 0.0005009 [0.0000368, 0.0009649] | 5.31 [0.39, 10.24] | **0.034** |
| Any potentially preventable admissions | 0.010 (0.110) | 0.0005167 [0.000131, 0.0009023] | 5.17 [1.31, 9.02] | **0.009** |
| # of potentially preventable admissions for chronic conditions | 0.0060663 (0.0776516) | 0.0002991 [-0.0000878, 0.000686] | 4.93 [-1.45, 11.30] | 0.130 |
| Any potentially preventable admissions for chronic conditions | 0.007 (0.089) | 0.0003395 [0.0000207, 0.0006584] | 4.85 [2.96, 9.41] | **0.037** |
| **Emergency Department Use** | | | | |
| # of all-cause ED visits | 0.1034144 (0.3045055) | -0.0000276 [-0.0022935, 0.0022384] | -0.03 [-2.21, 2.16] | 0.981 |
| Any all-cause ED visit | 0.147 (0.597) | 0.0005886 [-0.0006213, 0.0017985] | 0.40 [-0.42, 1.22] | 0.340 |
| **Patient Composition** | | | | |
| Age | 73.7 (9.8) | 0.0879957 [0.0485583, 0.127433] | 0.12 [0.07, 0.17] | **<0.001** |
| Proportion Female | 0.557 (0.50) | 0.0012816 [-0.0006954, 0.0032586] | 0.23 [-0.12, 0.59] | 0.204 |
| Proportion White | 0.863 (0.34) | 0.0001697 [-0.0010065, 0.0013459] | 0.02 [-0.12, 0.16] | 0.777 |
| HCC score | 1.27 (1.24) | 0.0115599 [0.0032683, 0.0198515] | 0.91 [0.26, 1.56] | **0.006** |

Notes/Sources: Unadjusted and adjusted differential changes in outcome variables are averaged at the patient-quarter level for PE patients and matched controls. Adjusted regression coefficients are estimated using a stacked difference-in-differences model that includes **physician and time fixed effects**. Standard errors are clustered at the level of the assigned primary care physician. Adjusted percentage differential change is calculated by dividing the adjusted differential change obtained from the difference-in-differences regression, by the pre-acquisition mean for PE-acquired physicians.

© 2026 Dixit MN et al. JAMA Health Forum.

## eTable 7. Differential change in natural log transformed outcomes of interest, PE and matched controls, 2016-2022

| | Pre-acquisition mean (SD) | DiD [95% C.I.] | DiD % | p-value |
|---|---|---|---|---|
| **Hospitalizations** | | | | |
| # of all-cause admissions | 0.067 (0.317) | -0.0014782 [-0.0037768, 0.0008203] | -0.001 [-0.004, 0.0008] | 0.207 |
| # of potentially preventable admissions | 0.010 (0.111) | 0.0001103 [-0.000826, 0.0010466] | 0.0001 [-0.0008, 0.001] | 0.817 |
| # of potentially preventable admissions for chronic conditions | 0.007 (0.089) | -0.0000858 [-0.000855, 0.0006834] | -0.00009 [-0.0009, 0.0007] | 0.827 |
| **Emergency Department Use** | | | | |
| # of all-cause ED visits | 0.150 (0.601) | -0.0030087 [-0.0060545, 0.0000371] | -0.003 [-0.006, 0.00004] | 0.053 |
| **Patient Composition** | | | | |
| Age | 73.6 (9.9) | 0.0000977 [0.0000564, 0.0001391] | 0.0001 [0.00006, 0.0001] | **<0.001** |
| HCC score | 1.284 (1.256) | -0.0005637 [-0.0038994, 0.002772] | -0.0006 [-0.004, 0.003] | 0.740 |

Notes/Sources: Unadjusted and adjusted differential changes in outcome variables are averaged at the patient-quarter level for PE patients and matched controls. Outcomes were transformed using ln(y + *c*) where $c = 0.1$ to avoid having rows with zeroes becoming undefined. Adjusted regression coefficients include beneficiary, physician, and time fixed effects. Standard errors are clustered at the level of the assigned primary care physician. Adjusted percentage differential change is calculated using $e^{\beta} - 1$ where $\beta$ is the adjusted differential change obtained from the difference-in-differences regression.

© 2026 Dixit MN et al. JAMA Health Forum.

**eTable 8. Differential change in outcomes of interest with alternative matching strategy, PE and matched controls, 2016-2022**

| | Pre-acquisition mean (SD) | DiD [95% C.I.] | DiD % | p-value |
|---|---|---|---|---|
| **Hospitalizations** | | | | |
| # of all-cause admissions | 0.0534082 (0.2248507) | -0.0023339 [-0.00365, -0.0010178] | -3.48 [-5.45, -1.52] | **0.001** |
| Any all-cause admissions | 0.067 (0.315) | -0.0016404 [-0.0025282, -0.0007526] | -3.05 [-4.70, -1.40] | **<0.001** |
| # of potentially preventable admissions | 0.0094274 (0.096638) | -0.0002106 [-0.0006569, 0.0002357] | -2.11 [-6.57, 2.36] | 0.355 |
| Any potentially preventable admissions | 0.010 (0.110) | -0.0002464 [-0.0006202, 0.0001275] | -2.55 [-6.42, 1.32] | 0.196 |
| # of potentially preventable admissions for chronic conditions | 0.0060663 (0.0776516) | -0.0001339 [-0.0005047, 0.000237] | -1.91 [-7.21, 3.39] | 0.479 |
| Any potentially preventable admissions for chronic conditions | 0.007 (0.089) | -0.0001297 [-0.0004368, 0.0001774] | -0.21 [-0.71, 0.29] | 0.408 |
| **Emergency Department Use** | | | | |
| # of all-cause ED visits | 0.1034144 (0.3045055) | -0.0040344 [-0.0060941, -0.0019746] | -2.67 [-4.04, -1.31] | **< 0.001** |
| Any all-cause ED visit | 0.147 (0.597) | -0.0023963 [-0.0035361, -0.0012566] | -2.28 [-3.36, -1.20] | **< 0.001** |
| **Patient Composition** | | | | |
| Age | 73.7 (9.8) | 0.000007 [-0.0003691 0.0003833] | 0.000009 [-0.0005, 0.0005] | 0.970 |
| Proportion Female | 0.557 (0.50) | -0.0009463 [-0.0028563, 0.0009637] | -0.17 [-0.51, 0.17] | 0.332 |
| Proportion White | 0.863 (0.34) | 0.0012229 [-0.0000637, 0.0025094] | 0.14 [-0.01, 0.30] | 0.062 |
| HCC score | 1.27 (1.24) | -0.0040617 [-0.0107257, 0.0026023] | -0.32 [-0.84, 0.20] | 0.232 |

Notes/Sources: Patients linked to physicians who eventually are PE acquired were matched to five control patients using an exact match for state and one standard deviation caliper match on baseline HCC score. Unadjusted and adjusted differential changes in outcome variables are averaged at the patient-quarter level for PE patients and matched controls. Adjusted regression coefficients are estimated using a stacked difference-in-differences model that includes beneficiary, physician, and time fixed effects except for proportion female and proportion White which exclude beneficiary fixed effects. Standard errors are clustered at the level of the assigned primary care physician.

© 2026 Dixit MN et al. JAMA Health Forum.

**eTable 9. Differential change in outcomes of interest with randomized acquisition quarters for controls, PE and all controls, 2016-2022**

| | Pre-acquisition mean (SD) | DiD [95% C.I.] | DiD % | p-value |
|---|---|---|---|---|
| **Hospitalizations** | | | | |
| # of all-cause admissions | 0.0534082 (0.2248507) | -0.0012347 [-0.003023, 0.0005536] | -2.31 [-5.66, 1.04] | 0.176 |
| Any all-cause admissions | 0.067 (0.315) | -0.000817 [-0.001991, 0.0003569] | -1.22 [-2.97, 0.53] | 0.173 |
| # of potentially preventable admissions | 0.0094274 (0.096638) | -0.0006487 [-0.001209, -0.0000883] | -6.88 [-12.82, -0.94] | **0.023** |
| Any potentially preventable admissions | 0.010 (0.110) | -0.0005339 [-0.0009992, -0.0000686] | -5.34 [-9.99, -0.69] | **0.025** |
| # of potentially preventable admissions for chronic conditions | 0.0060663 (0.0776516) | -0.000512 [-0.0009638, -0.0000603] | -8.44 [-15.89, -0.99] | **0.026** |
| Any potentially preventable admissions for chronic conditions | 0.007 (0.089) | -0.0004153 [-0.0007891, -0.0000415] | -5.93 [-11.27, -0.59] | **0.029** |
| **Emergency Department Use** | | | | |
| # of all-cause ED visits | 0.1034144 (0.3045055) | -0.0005394 [-0.0032373, 0.0021586] | -0.52 [-3.13, 2.09] | 0.695 |
| Any all-cause ED visit | 0.147 (0.597) | -0.0001518 [-0.0015943, 0.0012907] | -0.10 [-1.08, 0.88] | 0.837 |
| **Patient Composition** | | | | |
| Age | 73.7 (9.8) | 0.0004214 [-0.0000312, 0.0008741] | 0.0006 [-0.00004, 0.001] | 0.068 |
| Proportion Female | 0.557 (0.50) | 0.0005245 [-0.0025031, 0.0035521] | 0.09 [-0.46, 0.64] | 0.734 |
| Proportion White | 0.863 (0.34) | -0.0016429 [-0.0039255, 0.0006396] | -0.19 [-0.45, 0.07] | 0.158 |
| HCC score | 1.27 (1.24) | 0.0614982 [0.0496926, 0.0733038] | 4.84 [3.91, 5.77] | **<0.001** |

Notes/Sources: Unadjusted and adjusted differential changes in outcome variables are averaged at the patient-quarter level for PE patients and all controls. Adjusted regression coefficients are estimated using a stacked difference-in-differences model that includes beneficiary, physician, and time fixed effects except for proportion female and proportion White which exclude beneficiary fixed effects. Standard errors are clustered at the level of the assigned primary care physician. Adjusted percentage differential change is calculated by dividing the adjusted differential change obtained from the difference-in-differences regression, by the pre-acquisition mean for PE-acquired physicians.

© 2026 Dixit MN et al. JAMA Health Forum.

**eTable 10. Differential change in outcomes of interest, PE and matched controls with acquisition dates prior to 2020, 2016-2022**

| | Pre-acquisition mean (SD) | DiD [95% C.I.] | DiD % | p-value |
|---|---|---|---|---|
| **Hospitalizations** | | | | |
| # of all-cause admissions | 0.080 (0.367) | -0.0020622 [-0.0059725, 0.001848] | -2.58 [-7.47, 2.31] | 0.301 |
| Any all-cause admissions | 0.059 (0.235) | -0.0013322 [-0.0039473, 0.001283] | -2.26 [-6.69, 2.17] | 0.318 |
| # of potentially preventable admissions | 0.013 (0.141) | -0.001132 [-0.0025147, 0.0002508] | -8.71 [-19.34, 1.93] | 0.109 |
| Any potentially preventable admissions | 0.010 (0.100) | -0.0008495 [-0.0019358, 0.0002369] | -8.50 [-19.36, 2.37] | 0.125 |
| # of potentially preventable admissions for chronic conditions | 0.009 (0.117) | -0.0011298 [-0.0023393, 0.0000796] | -12.55 [-25.99, 0.88] | 0.067 |
| Any potentially preventable admissions for chronic conditions | 0.008 (0.088) | -0.0007026 [-0.0016198 0.0002146] | -8.78 [-20.25, 26.83] | 0.133 |
| **Emergency Department Use** | | | | |
| # of all-cause ED visits | 0.175 (0.621) | -0.0040733 [-0.0099303, 0.0017837] | -2.33 [-5.67, 1.02] | 0.173 |
| Any all-cause ED visit | 0.114 (0.318) | -0.0014975 [-0.0048179, 0.0018229] | -1.31 [-4.23, 1.60] | 0.377 |
| **Patient Composition** | | | | |
| Age | 72.3 (10.7) | 0.0014836 [0.0003936, 0.0025736] | 0.002 [0.0005, 0.003] | **0.008** |
| Proportion Female | 0.560 (0.50) | -0.0023525 [-0.0068638, 0.0021589] | -0.42 [-1.23, 0.39] | 0.307 |
| Proportion White | 0.824 (0.381) | -0.0004493 [-0.0031793, 0.0022807] | -0.05 [-0.39, 0.28] | 0.747 |
| HCC score | 1.19 (1.24) | -0.0129728 [-0.0299363, 0.0039908] | -1.09 [-2.52, 0.34] | 0.134 |

Notes/Sources: Unadjusted and adjusted differential changes in outcome variables are averaged at the patient-quarter level for PE patients and matched controls. PE patients, and matched controls, whose PCP's were acquired after 2019 were excluded from this sample to examine the effect of a longer post-period on the outcomes. Adjusted regression coefficients are estimated using a stacked difference-in-differences model that includes beneficiary, physician, and time fixed effects except for proportion female and proportion White which exclude beneficiary fixed effects. Standard errors are clustered at the level of the assigned primary care physician.

© 2026 Dixit MN et al. JAMA Health Forum.

**eTable 11. Differential change in outcomes of interest, PE and matched controls, 2016-2022 (Callaway and Sant'Anna estimator)**

| | DiD [95% C.I.] | p-value |
|---|---|---|
| **Hospitalizations** | | |
| # of all-cause admissions | -0.0216 [-0.0373, -0.0060] | 0.007 |
| # of potentially preventable admissions | -0.00017 [-0.0026, 0.0023] | 0.890 |
| # of potentially preventable admissions for chronic conditions | -0.00021 [-0.0018, 0.0014] | 0.797 |
| **Emergency Department Use** | | |
| # of all-cause ED visits | -0.021 [-0.0952, 0.0530] | 0.570 |
| **Patient Composition** | | |
| Age | .000295 [-0.0022, 0.0028] | 0.822 |
| Proportion Female | .00006 [-0.0001, 0.0002] | 0.57 |
| Proportion White | -.00697 [-0.0152, 0.0012] | 0.09 |
| HCC Score | .0395 [0.0077, 0.0715] | **0.02** |

Notes/Sources: DiD regression coefficients are estimated using the Callaway & Sant'Anna estimator using never acquired units as controls.

© 2026 Dixit MN et al. JAMA Health Forum.

**eTable 12. Differential change in outcomes of interest without physician fixed effects, PE and matched controls, 2016-2022**

| | Pre-acquisition mean (SD) | DiD [95% C.I.] | DiD % | p-value |
|---|---|---|---|---|
| **Hospitalizations** | | | | |
| # of all-cause admissions | 0.0534082 (0.2248507) | -0.0009818 [-0.0022246, 0.0002611] | -1.84 [-4.17, 0.49] | 0.122 |
| Any all-cause admission | 0.067 (0.315) | -0.0004586 [-0.0013017, 0.0003845] | -0.68 [-1.94, 0.57] | 0.286 |
| # of potentially preventable admissions | 0.0094274 (0.096638) | -0.0000194 [-0.0004457, 0.0004069] | -0.21 [-4.73, 4.32] | 0.929 |
| Any potentially preventable admissions | 0.010 (0.110) | 0.0000456 [-0.0003128, 0.000404] | 0.46 [-3.13, 4.04] | 0.803 |
| # of potentially preventable admissions for chronic conditions | 0.0060663 (0.0776516) | -0.000064 [-0.0004155, 0.0002875] | -1.06 [-6.85, 4.74] | 0.721 |
| Any potentially preventable admissions for chronic conditions | 0.007 (0.089) | 0.0000113 [-0.0002832, 0.0003057] | 0.16 [-4.05, 4.37] | 0.940 |
| **Emergency Department Use** | | | | |
| # of all-cause ED visits | 0.1034144 (0.3045055) | -0.0025365 [-0.0044889, -0.000584] | -2.45 [-4.34, -0.56] | **0.011** |
| Any all-cause ED visit | 0.147 (0.597) | -0.0010915 [-.0021741, -0.000009] | -0.74 [-1.48, -0.01] | **0.048** |
| **Patient Composition** | | | | |
| Age | 73.7 (9.8) | 0.0002563 [-.0001237, 0.0006363] | 0.0003 [-0.0002, 0.0009] | 0.186 |
| Proportion Female | 0.557 (0.50) | 0.0019211 [-0.0008449 0.0046871] | 0.34 [-0.15, 0.84] | 0.173 |
| Proportion White | 0.863 (0.34) | 0.0115439 [0.0092949 0.0137929] | 1.34 [1.08, 1.60] | **<0.001** |
| HCC score | 1.27 (1.24) | -0.0083593 [-0.014915, -0.0018036] | -0.66 [-1.17, -0.14] | **0.012** |

Notes/Sources: Unadjusted and adjusted differential changes in outcome variables are averaged at the patient-quarter level for PE patients and matched controls. Adjusted regression coefficients are estimated using a stacked difference-in-differences model that includes **beneficiary and time fixed effects** except for proportion female and proportion White which only include time fixed effects. Standard errors are clustered at the level of the assigned primary care physician. Adjusted percentage differential change is calculated by dividing the adjusted differential change obtained from the difference-in-differences regression, by the pre-acquisition mean for PE-acquired physicians.

© 2026 Dixit MN et al. JAMA Health Forum.

**eTable 13. Differential change in outcomes of interest, PE and matched controls, 2016-2022 (including patientXphysician FE)**

| | Pre-acquisition mean (SD) | DiD [95% C.I.] | p-value |
|---|---|---|---|
| **Hospitalizations** | | | |
| # of all-cause admissions | 0.067 (0.315) | -0.0009 [-0.0023, 0.0003] | 0.153 |
| # of potentially preventable admissions | 0.010 (0.110) | 0.00008 [-0.00038, 0.00055] | 0.725 |
| # of potentially preventable admissions for chronic conditions | 0.007 (0.089) | -0.00001 [-0.00040, 0.00036] | 0.920 |
| **Emergency Department Use** | | | |
| # of all-cause ED visits | 0.147 (0.597) | -0.00277 [-0.0048, -0.00066] | **0.010** |
| **Patient Composition** | | | |
| Age | 73.7 (9.8) | 0.0009 [-0.0011, 0.0029] | 0.377 |
| Proportion Female | 0.557 (0.50) | 0.0001 [-0.0001, 0.0001] | 0.807 |
| Proportion White | 0.863 (0.34) | -0.0007 [-0.0024, 0.0010] | 0.426 |
| HCC Score | 1.27 (1.24) | -0.00532 [-0.0123, 0.00173] | 0.139 |

Notes/Sources: DiD regression coefficients are estimated using a stacked difference-in-differences model that includes patient, physician, patientXphysician and time fixed effects. Standard errors are clustered at the level of the assigned primary care physician. Adjusted percentage differential change is calculated by dividing the adjusted differential change obtained from the difference-in-differences regression, by the pre-acquisition mean for PE-acquired physicians.

© 2026 Dixit MN et al. JAMA Health Forum.

**eTable 14. Differential change in outcomes of interest, PE and matched controls, 2016-2022 (balanced panel)**

| | Pre-acquisition mean (SD) | DiD [95% C.I.] | p-value |
|---|---|---|---|
| **Hospitalizations** | | | |
| # of all-cause admissions | 0.067 (0.315) | .00001 [-0.00199, 0.0020] | 0.987 |
| # of potentially preventable admissions | 0.010 (0.110) | 0.00019 [-0.00041, 0.00081] | 0.527 |
| # of potentially preventable admissions for chronic conditions | 0.007 (0.089) | 0.00005 [-0.00045, 0.00056] | 0.832 |
| **Emergency Department Use** | | | |
| # of all-cause ED visits | 0.147 (0.597) | -0.00323 [-0.00642, 0.0000] | **0.048** |
| **Patient Composition** | | | |
| Age | 73.7 (9.8) | -0.0001 [-0.0066, 0.0035] | 0.549 |
| Proportion Female | 0.557 (0.50) | -0.0001 [-0.0003, 0.0000] | 0.59 |
| Proportion White | 0.863 (0.34) | 0.00001 [-0.0002, 0.0002] | 0.914 |
| HCC Score | 1.27 (1.24) | 0.0061 [-0.0061, 0.0183] | 0.327 |

Notes/Sources: DiD regression coefficients are estimated using a stacked difference-in-differences model that includes patient, physician, and time fixed effects. In this specification, we fully balance the panel to limit the sample to beneficiaries attributed to the same PCP over the entire sample, i.e., the sample is limited to patients that are attributed to the same PCP for all the 7 years in the data. Standard errors are clustered at the level of the assigned primary care physician. Adjusted percentage differential change is calculated by dividing the adjusted differential change obtained from the difference-in-differences regression, by the pre-acquisition mean for PE-acquired physicians.

© 2026 Dixit MN et al. JAMA Health Forum.

## eTable 15. Summary of outcomes across specifications

| | Base Model | Woolridge DID | Without Bene FE | Log (outcome) | Alt Matching | All Controls | Pre-2020 Deals | Without NPI FE | With NPIXBene FE | Balanced Panel | CS |
|---|---|---|---|---|---|---|---|---|---|---|---|
| **Hospitalizations** | | | | | | | | | | | |
| # of all-cause admissions | No change | No change | No change | No change | – | No change | No change | No change | No change | No change | – |
| # of potentially preventable admissions | No change | No change | + | No change | No change | – | No change | No change | No change | No change | No change |
| # of potentially preventable admissions for chronic conditions | No change | No change | No change | No change | No change | – | No change | No change | No change | No change | No change |
| **Emergency Department Use** | | | | | | | | | | | |
| # of all-cause ED visits | – | – | No change | – | – | No change | No change | – | – | – | No change |
| **Patient Composition** | | | | | | | | | | | |
| Age | No change | No change | + | + | No change | No change | + | No change | No change | No change | No change |
| Proportion Female | No change | No change | No change | No change | No change | No change | No change | No change | No change | No change | No change |
| Proportion White | No change | No change | No change | No change | No change | No change | No change | + | No change | No change | No change |
| HCC score | No change | No change | + | No change | No change | + | No change | – | No change | No change | + |

Notes/Sources: For detailed results, see respective appendix tables above. (+) denotes a statistically significant increase, (–) denotes a statistically significant decrease, and (No change) denotes no significant change at α= 0.05.

© 2026 Dixit MN et al. JAMA Health Forum.

**eFigure. Stacked event studies of outcomes of interest, PE and matched controls, 2016-2022**

Number of potentially avoidable hospitalizations (PQI 90)

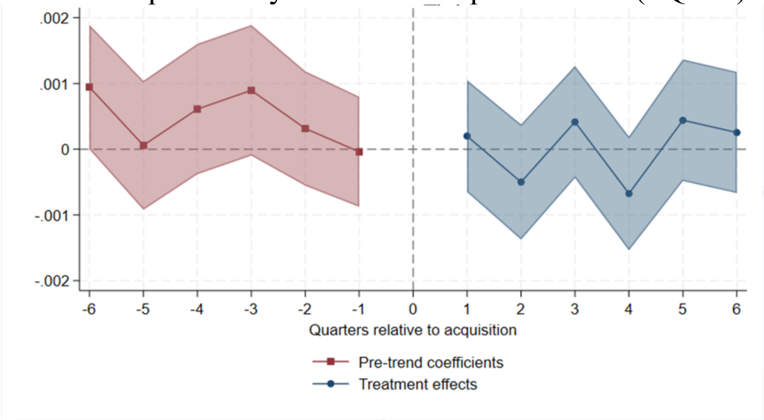


Number of potentially avoidable chronic illness related hospitalizations (PQI 92)

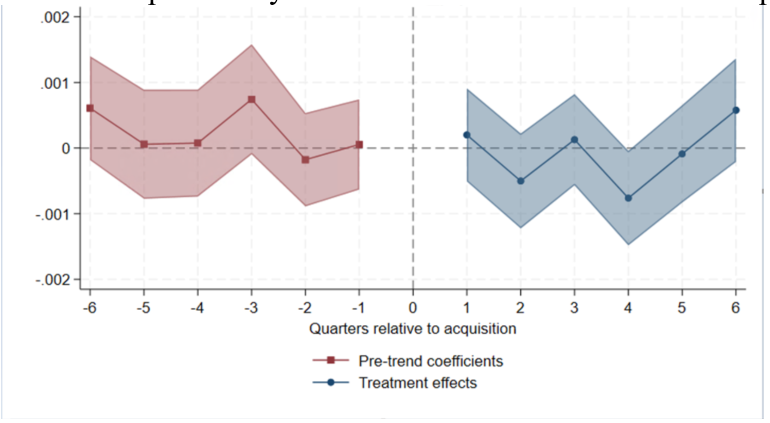


Mean age

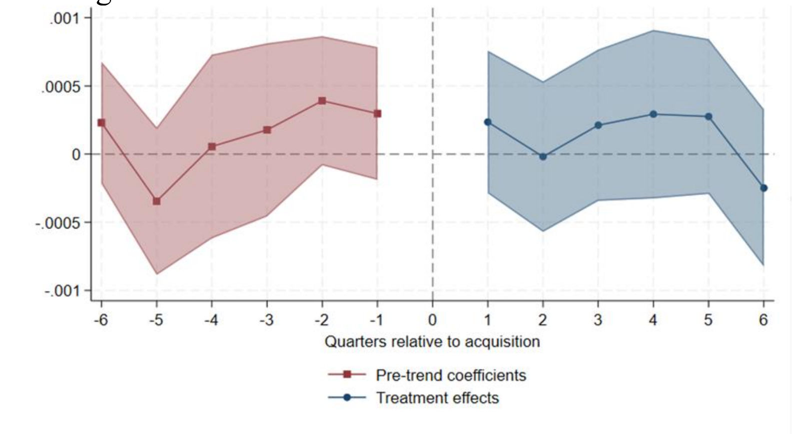


© 2026 Dixit MN et al. JAMA Health Forum.

Proportion female

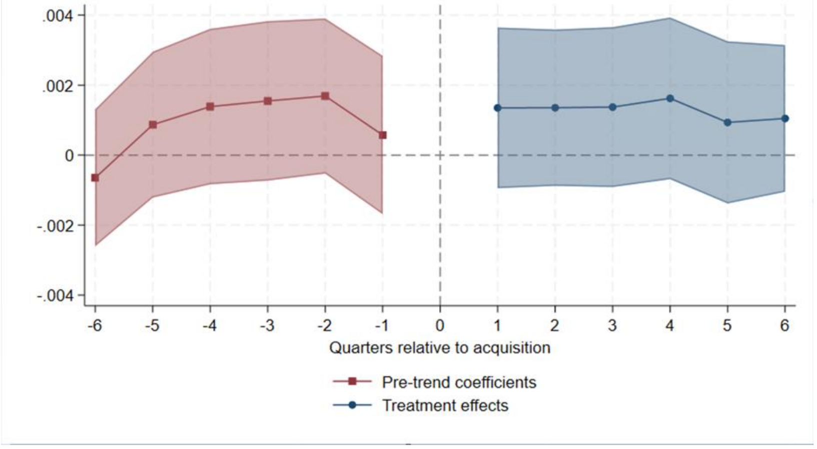


Proportion White

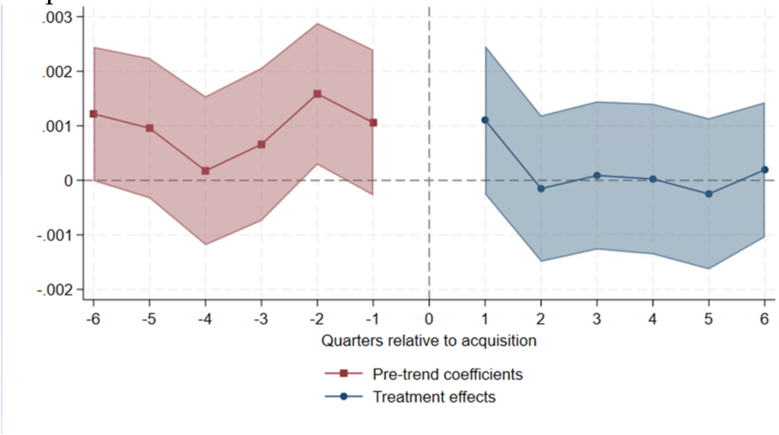


© 2026 Dixit MN et al. JAMA Health Forum.
