# Supplementary material for: Private Equity Acquisition in Primary Care and Avoidable Hospitalizations
Source: JAMA Health Forum. 2026 May 8;7(5):e261045. doi: 10.1001/jamahealthforum.2026.1045 (PMC13156794; doi:10.1001/jamahealthforum.2026.1045)
Supplement: Supplement 2. — Data Sharing Statement [file jamahealthforum-e261045-s002.pdf]

Data Sharing Statement

Dixit. Private Equity Acquisition in Primary Care and Avoidable Hospitalizations. *JAMA Health Forum*. Published May 08, 2026. doi:10.1001/jamahealthforum.2026.1045

Data
Data available: No
